# Supplementary material for: The developing mouse coronal suture at single-cell resolution
Source: Nat Commun. 2021 Aug 10;12:4797. doi: 10.1038/s41467-021-24917-9 (PMC8355337; doi:10.1038/s41467-021-24917-9)
Supplement: Supplementary file 1 — Supplementary Information [file 41467_2021_24917_MOESM1_ESM.pdf]

Supplementary Figure 1

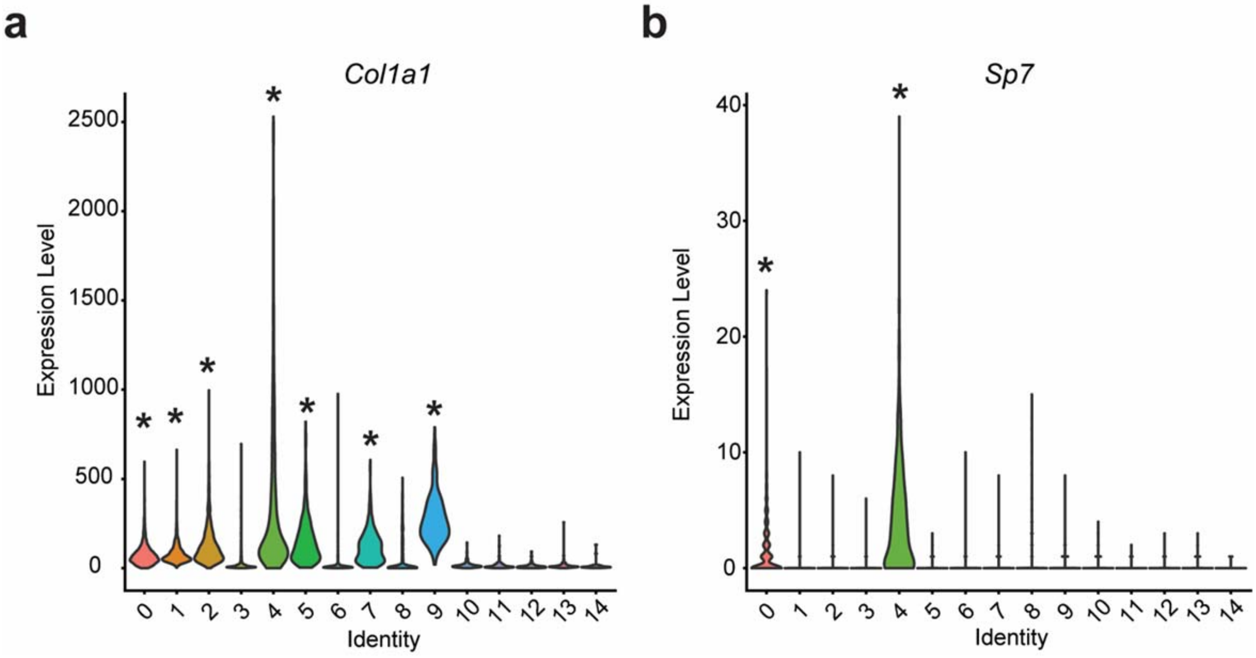

Supplementary Figure 1. Fibroblast and osteoblast markers label mesenchymal/osteogenic clusters. a Violin plot for *Col1a1*. **b** Violin plot for *Sp7*. Enriched clusters are marked by asterisks.

Supplementary Figure 2

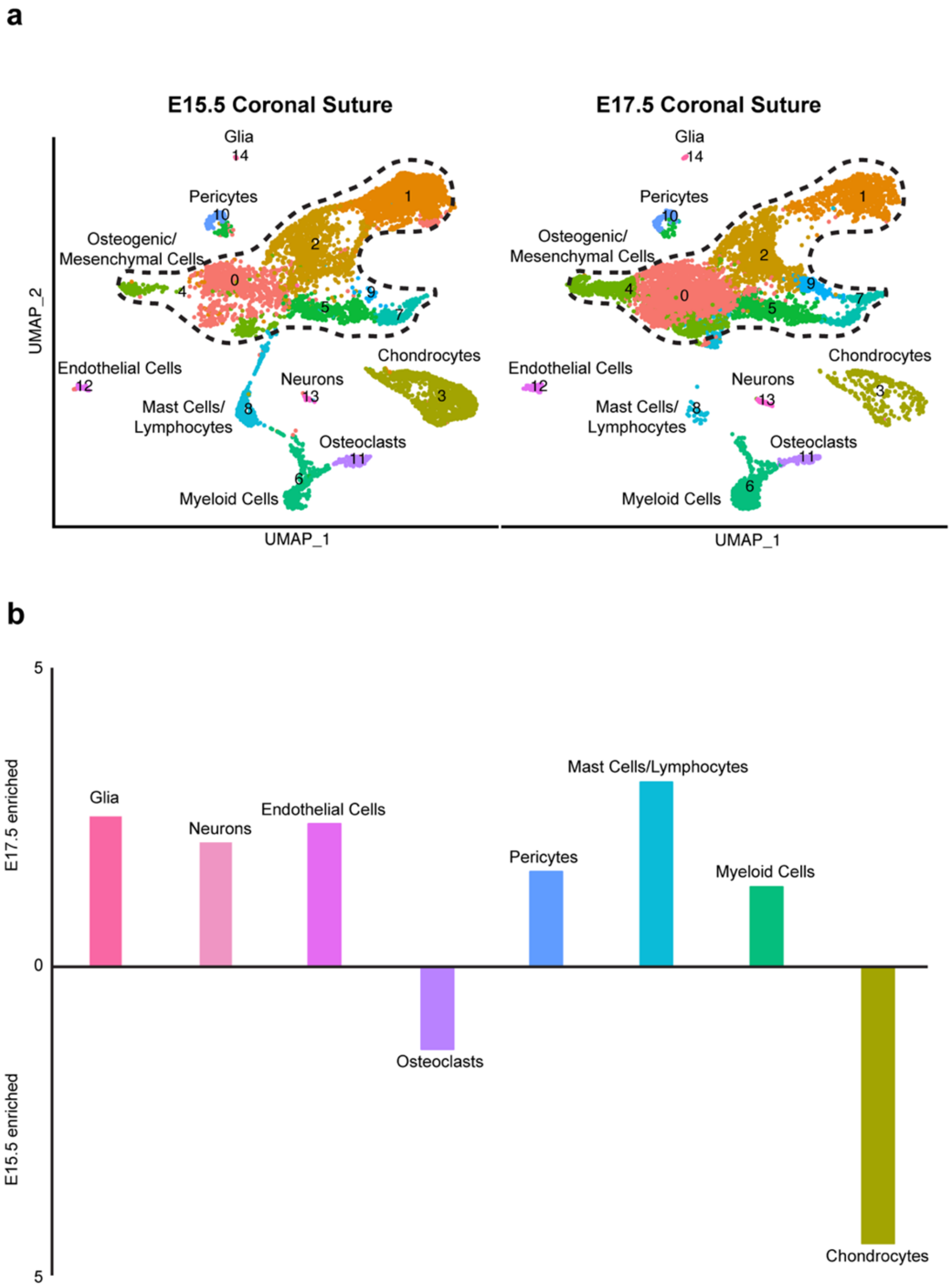

Supplementary Figure 2. Equivalent cell types are captured at E15.5 and E17.5. a UMAP plots separated by developmental stage. **b** Graphical depiction of the cluster proportions plotted as ratio between E15.5 and E17.5 cells within each cluster.

Supplementary Figure 3

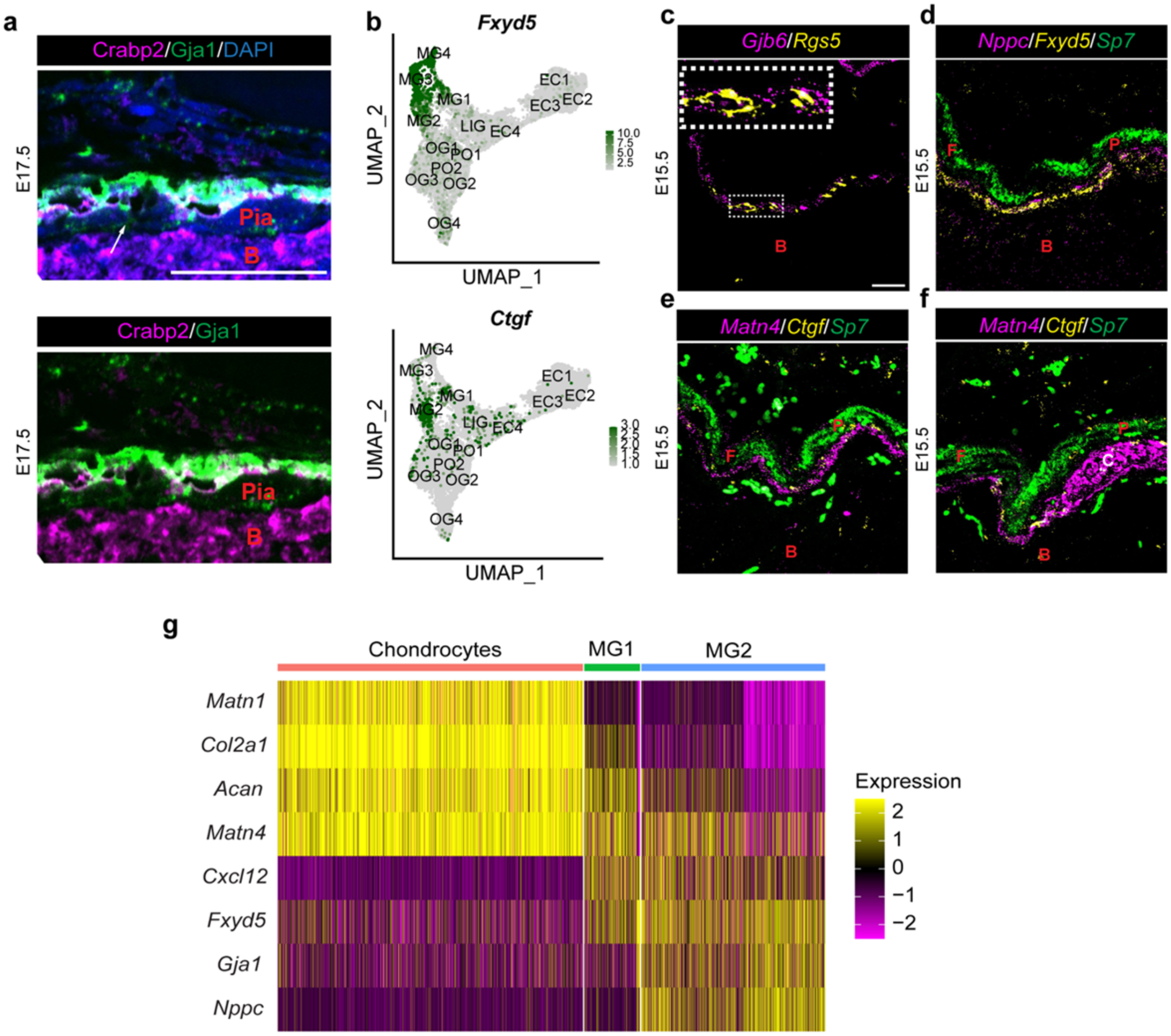

Supplementary Figure 3. Resolving meningeal subtypes. a Immunofluorescence of Crapb2 and Gja1 at E17.5. Scale bar = 25 µm. **b** Feature plots of genes validated by in situ experiments. **c-f** In situ analysis of coronal sutures for indicated markers at E15.5. **c** *Gjb6* and *Rgs5*. Dashed lines highlight magnified inset. **d** *Nppc and Fxyd5.* **e,f** *Matn4 and Ctgf. Sp7* marks the frontal (F) and parietal (P) bones in (**d-f**). **g** Heatmap of selected chondrocyte and dura mater markers. B, brain; C, cartilage. Immunofluorescent experiments were performed in triplicate. In situs were performed in biological triplicate. Scale bars = 50 µm.

Supplementary Figure 4

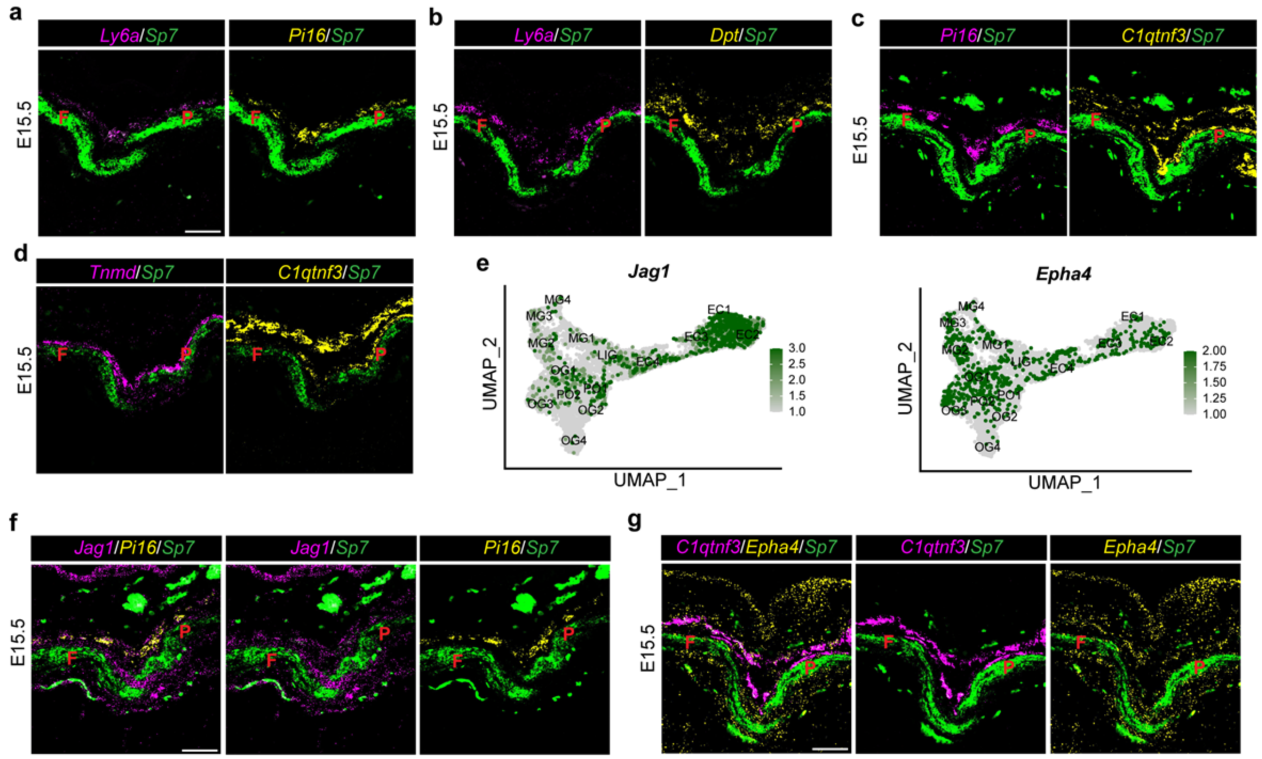

Supplementary Figure 4. Ectocranial layers overlay the coronal suture. a-d Single channel images of genes evaluated in Figure 3. **e** Feature plots of genes validated by in situ experiments. **f, g** In situ analysis of coronal sutures for indicated markers at E15.5. In situs were performed in biological triplicate. Scale bars = 50 µm.

Supplementary Figure 5

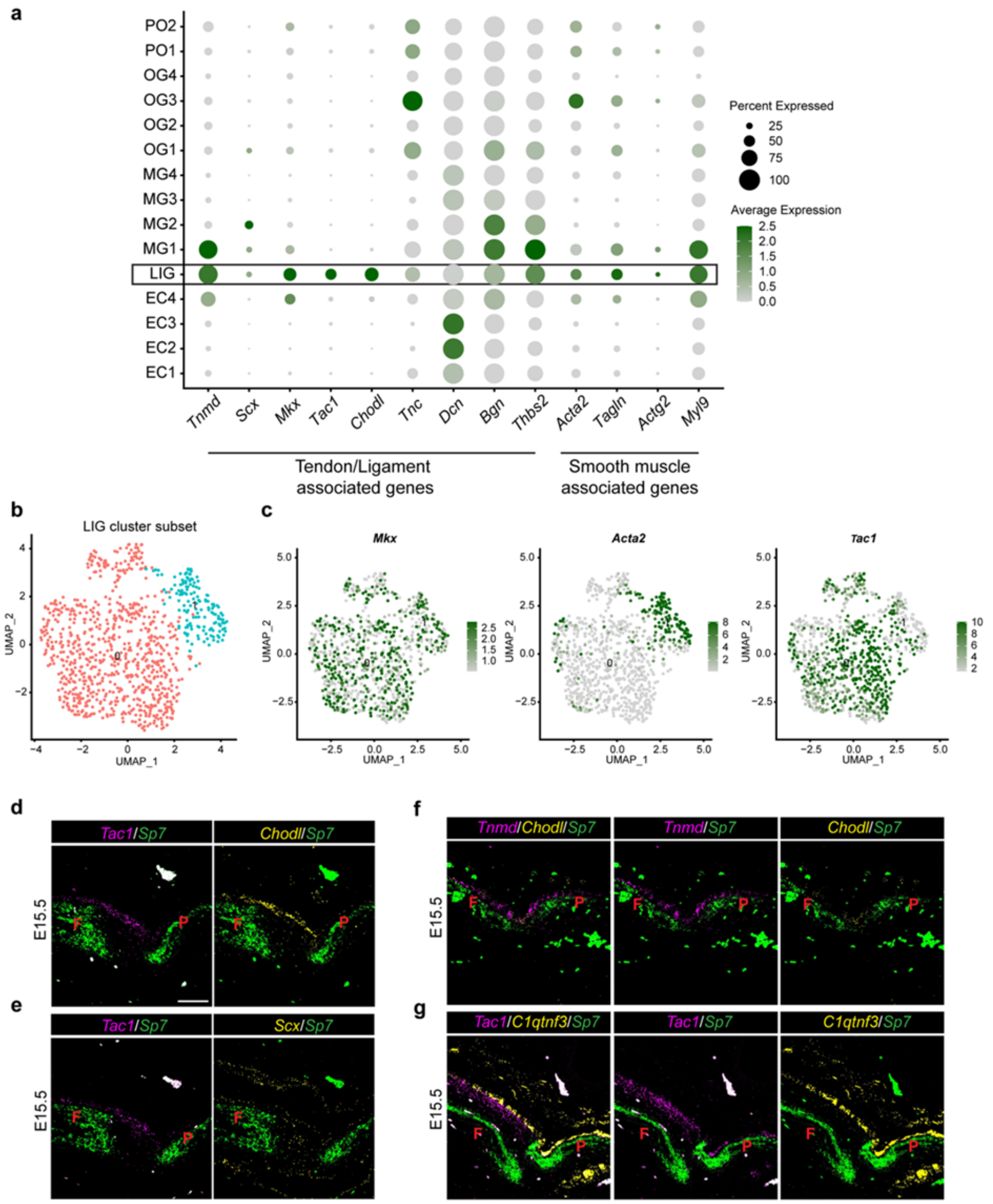

Supplementary Figure 5. LIG cluster can be divided into ligament-like and smooth muscle-like clusters. a Dot plot of markers associated with tendon/ligament identity and cellular contractility. **b** UMAP after re-clustering. **c** Feature plots of genes identified in LIG cluster. **d, e** Single channel images of genes evaluated in Figure 4. **f, g** In situ analysis for indicated markers at E15.5. *Sp7* marks the frontal (F) and parietal (P) bones in all in situs. In situs were performed in biological triplicate. Scale bar = 50 µm.

Supplementary Figure 6

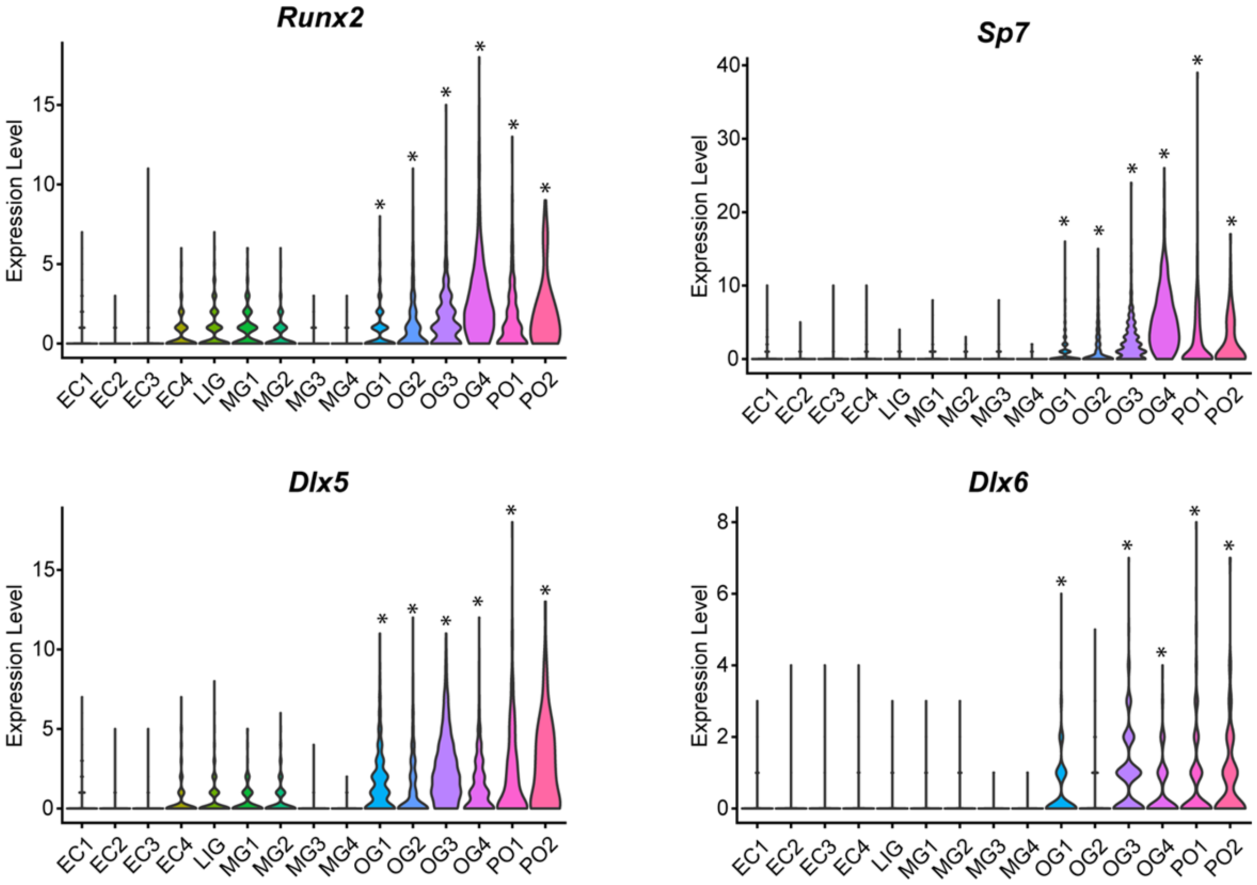

Supplementary Figure 6. Markers for the osteogenic subset. Violin plots for *Runx2, Sp7, Dlx5*, and *Dlx6*. Enriched clusters are marked by asterisks.

Supplementary Figure 7

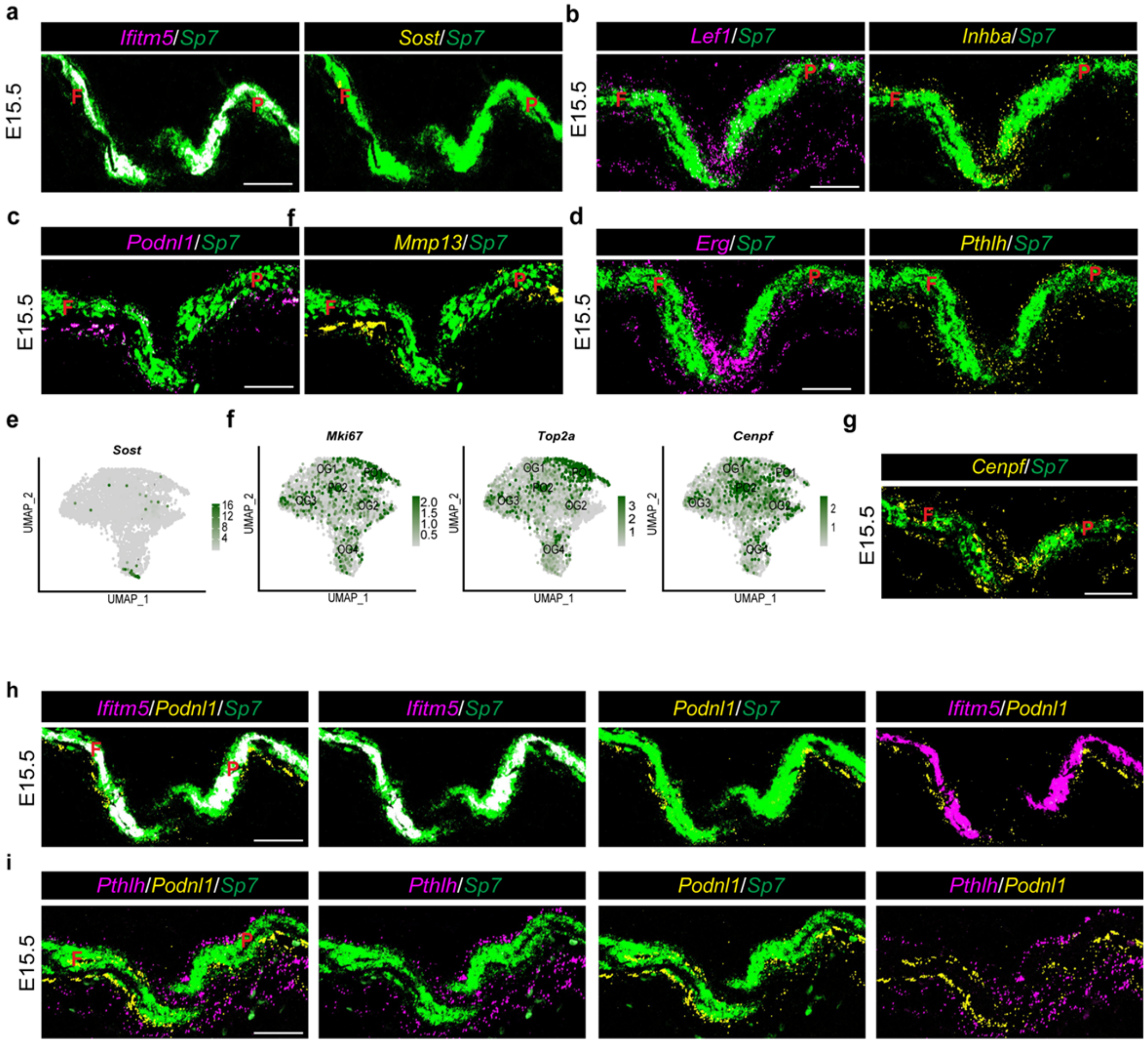

Supplementary Figure 7. In situ analysis of osteogenesis cluster markers. a-d Single channel images of genes evaluated in Figure 5. **e** Feature plot of *Sost.* **f** Feature plots of genes marking clusters PO1 and PO2. **g-i** In situ analysis of coronal sutures for indicated markers at E15.5. *Sp7* marks the frontal (F) and parietal (P) bones in all in situs. In situs were performed in biological triplicate. Scale bars = 50 µm.

Supplementary Figure 8

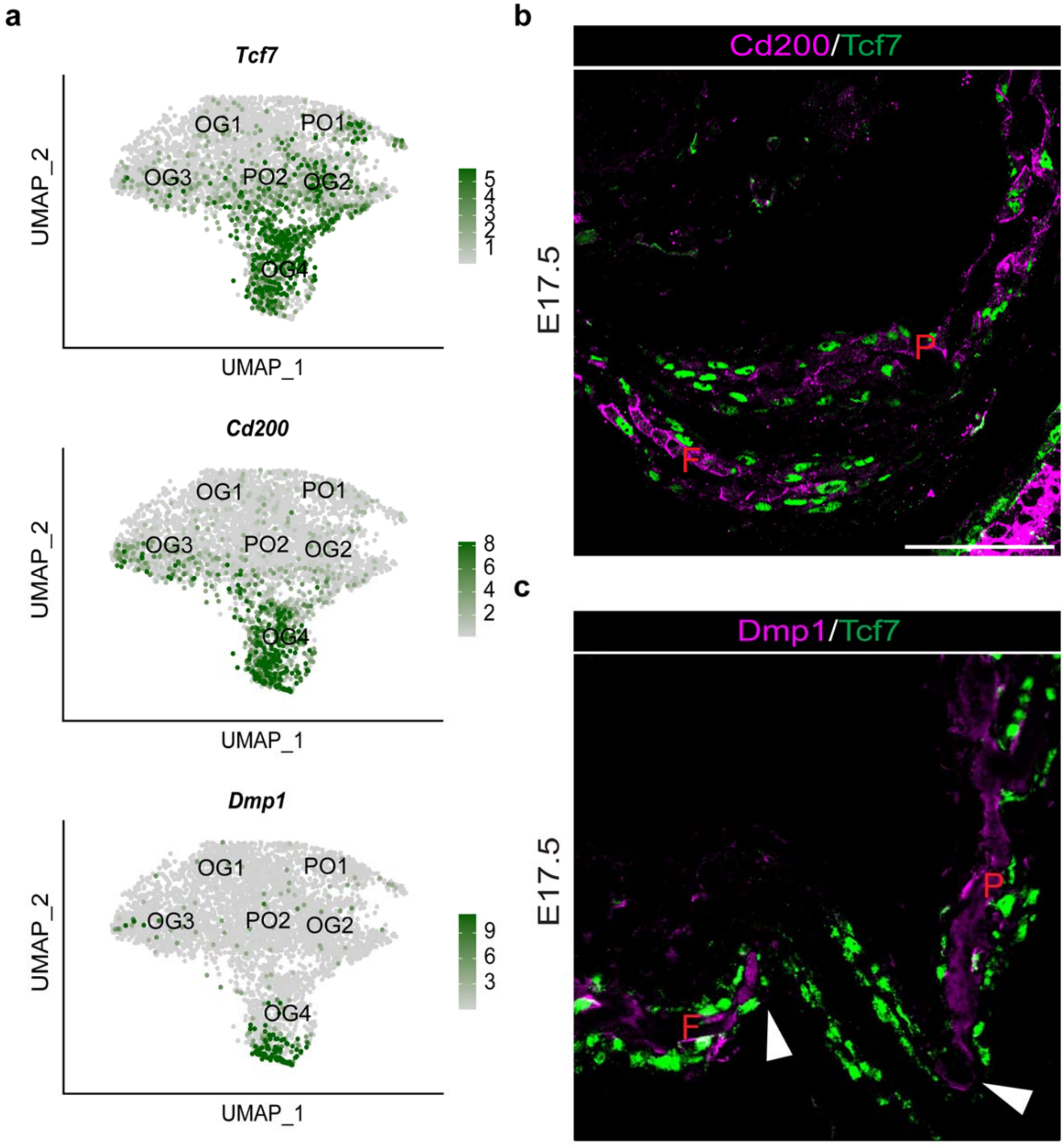

Supplementary Figure 8. Immunofluorescence analysis resolves osteogenic subtypes within the coronal suture. a Feature plots of genes validated by antibody staining. **b-c** Combinatorial antibody staining of coronal sutures for indicated markers at E17.5. Arrowheads mark bone fronts. F, Frontal bone; P, Parietal bone. Immunofluorescent experiments were performed in biological triplicate. Scale bars = 50 µm.

Supplementary Figure 9

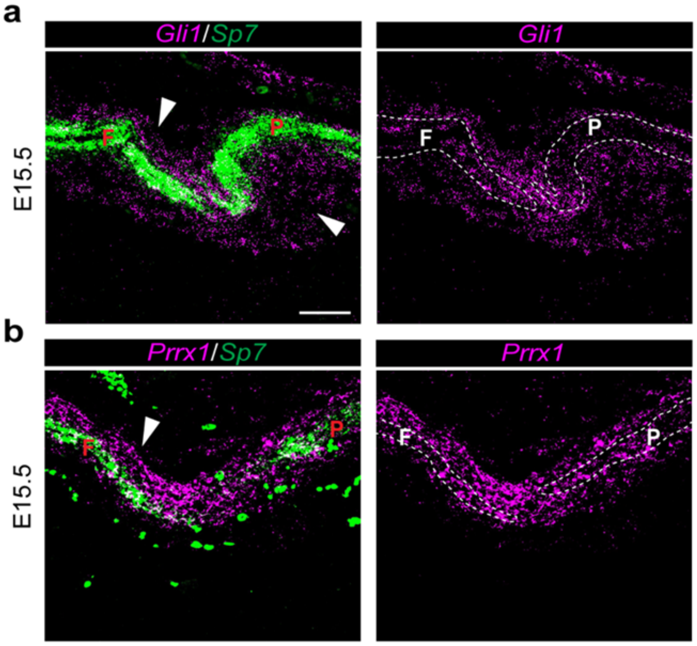

Figure 9. Asymmetric distribution of mesenchyme across the frontal and parietal bones. a-b In situ analysis of coronal sutures for indicated markers at E15.5. *Sp7* marks the frontal (F) and parietal (P) bones. Arrowheads indicate surfaces of bones where markers are enriched. Dashed lines mark bone. In situs were performed in biological triplicates. Scale bar = 50 µm.

Supplementary Figure 10

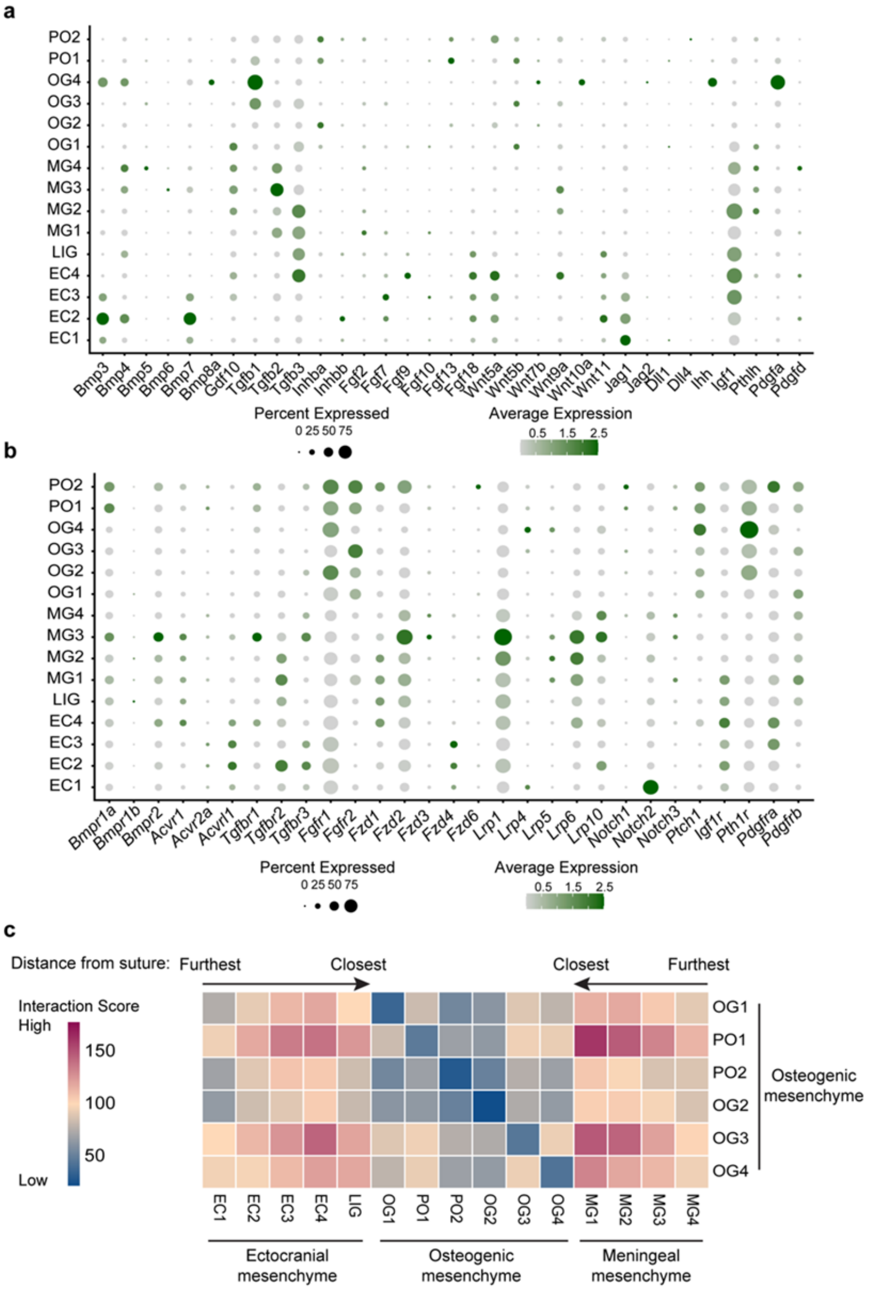

Supplementary Figure 10. Ligand and receptor expression and predicted interactions at the coronal suture. a Dot plot of a selected group of secreted factors expressed within the coronal suture. **b** Dot plot of a selected group of receptors expressed within the coronal suture. The size and color intensity of the dots shown in **a** and **b** correspond respectively to the percentage of cells within each cluster expressing the indicated gene, and the average expression level. **c** Heatmap of interaction scores between clusters from CellPhoneDB analysis. Ectocranial and meningeal clusters are arranged based on their validated distance from the coronal suture and bones. Heatmap scale shows counts of interactions.

Supplementary Figure 11

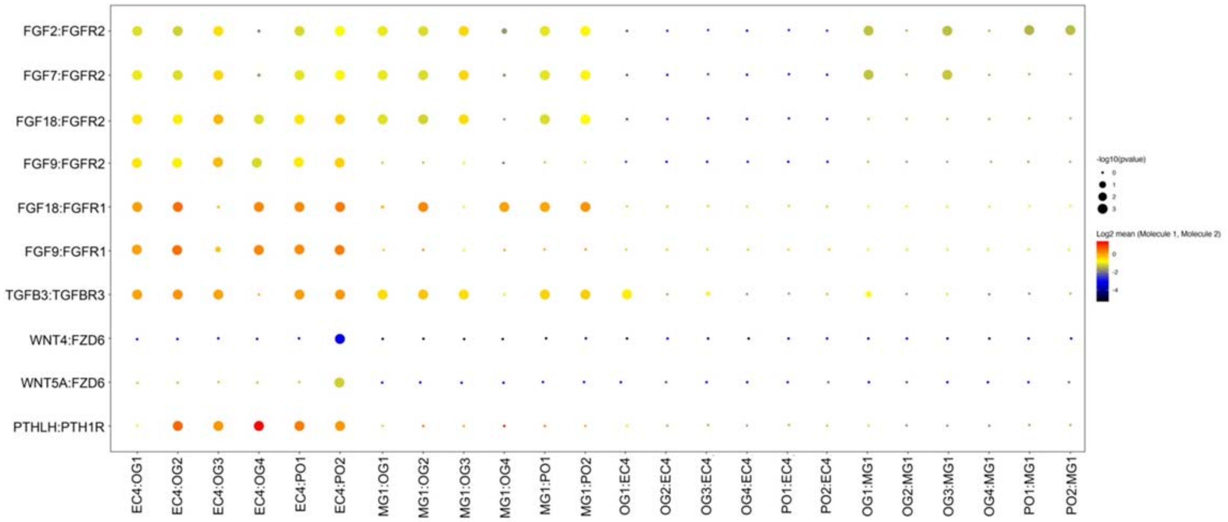

Supplementary Figure 11. Putative signaling interactions between osteogenic and non-osteogenic populations. Dot plot representing selected examples of ligand-receptor interactions between osteogenic and non- osteogenic clusters captured from CellPhone DB analysis. P values (one-sided) were calculated (as number of shuffled means > realmean) / (total of shuffled times)) following the workflow in: https://github.com/Teichlab/cellphonedb/blob/master/Docs/cluster_statistical_analysis_method_workflow.md. Adjustments were not made for multiple comparisons.

Supplementary Figure 12

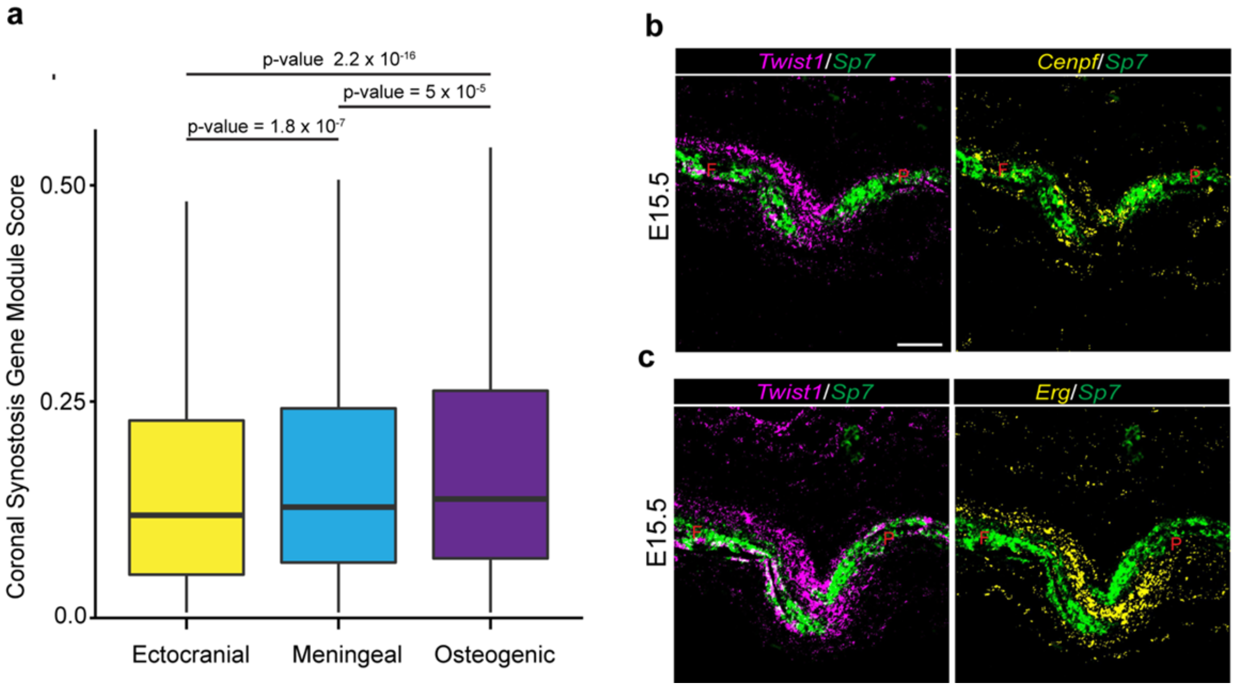

Supplementary Figure 12. Coronal synostosis genes are enriched in osteogenic cells. a Boxplot of coronal synostosis gene module scores from cells within ectocranial (n = 5550 biologically independent cells), meningeal (n = 1907 biologically independent cells), and osteogenic (n = 4712 biologically independent cells) clusters. The interquartile range (IQR) is defined by the box, the upper and lower borders of each box represent the first and third quartiles, the horizontal line within the box represents the median, and the whiskers represent 1.5*IQR from the first and third quartiles. P-value (two-sided pairwise Wilcoxson Rank Sum tests with Benjamini & Hochberg correction after Kruskal-Wallis test) are noted with black lines between boxplots. **b, c** Single channel images of genes evaluated in Figure 7. F, Frontal bone; P, Parietal bone. In situs were performed in biological replicates. Scale bars = 50 µm.

Supplementary Figure 13

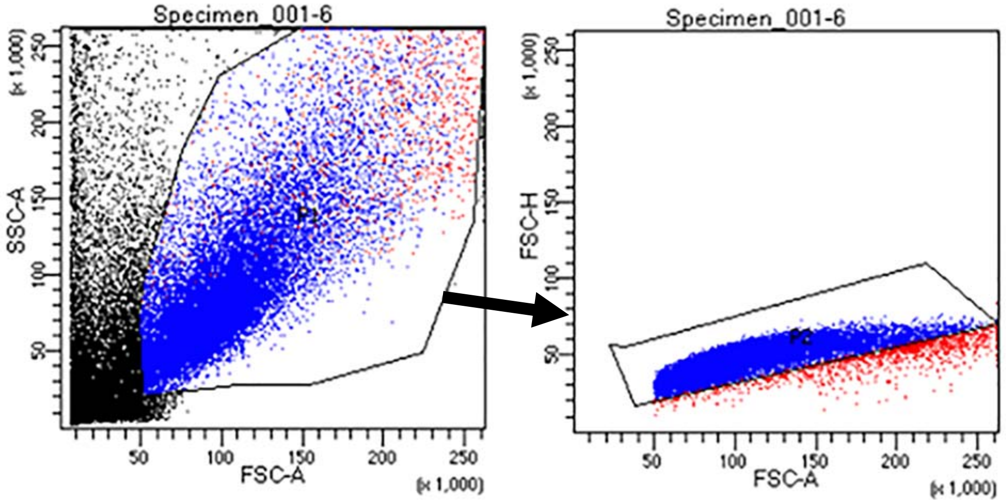

Tube: 6

Population	#Events	%Parent	%Total
All Events	40,792	####	100.0
P1	23,594	57.8	57.8
P2	22,092	93.6	54.2

Supplementary Figure 13. Gating strategy used to isolate single cells from E17.5 sutural material. Gating (P1 and P2) was used to purify live and individual cells prior to scRNA-seq.

Supplementary Data 1. Enriched gene list by cluster for integrated E15.5 and E17.5 datasets. Table of enriched genes for each cluster in the E15.5/E17.5 integrated dataset using the RNA assay.

Supplementary Data 2. Enriched gene list by cluster for integrated E15.5 and E17.5 osteogenic/mesenchymal subset. Table of enriched genes for each cluster in the osteogenic/mesenchymal subset using the RNA assay.

Supplementary Table 1. Genotyping Primers. Table of genotyping primers for mouse experiments.

Strain	Genotyping Primers	Primers (5'-3')
	Twist1 F	GGCTGTTTTCTATGACCGCT
Twist1 null	Twist1 R	AATCCATCTTGTTCAATGGCCGATC
	Tcf12 F	CTGGGACAGAAGTTCAGCACTTAGTAC
Tcf12 null	Tcf12 R	CATTCCTATACATCAGCTTCTTGGACG
	R26R_wt	GGAGCGGGAGAAATGGATATG
	R26R_com	AAAGTCGCTCTGAGTTGTTAT
Ai19	R26R_mut	GCGAAGAGTTTGTCCTCAACC
	Cre F	TGCTGTTTCACTGGTTATGCGG
Six2-Cre/Six-CreERT2	Cre R	TTGCCCCTGTTTCACTATCCAG
	15020	CTGAACTTGTGGCCGTTTAC
	24500	CAGGACAACGCCCACACA
	36178	ACACTTGCCTCTACCGGTTC
CAG-Sun1/sfGFP	oIMR9020	AAGGGAGCTGCAGTGGAGTA
