# Supplementary material for: The developing mouse coronal suture at single-cell resolution
Source: Nat Commun. 2021 Aug 10;12:4797. doi: 10.1038/s41467-021-24917-9 (PMC8355337; doi:10.1038/s41467-021-24917-9)
Supplement: Supplementary file 3 — Description of Additional Supplementary Files [file 41467_2021_24917_MOESM3_ESM.docx]

Description of Additional Supplementary Files

**Title: Supplementary Data 1. Enriched gene list by cluster for integrated E15.5 and E17.5 datasets.**

**Description:** Table of enriched genes for each cluster in the E15.5/E17.5 integrated dataset using the RNA assay.

**Title: Supplementary Data 2. Enriched gene list by cluster for integrated E15.5 and E17.5 osteogenic/mesenchymal subset.**

**Description:** Table of enriched genes for each cluster in the osteogenic/mesenchymal subset using the RNA assay.
