# Supplementary material for: The developing mouse coronal suture at single-cell resolution
Source: Nat Commun. 2021 Aug 10;12:4797. doi: 10.1038/s41467-021-24917-9 (PMC8355337; doi:10.1038/s41467-021-24917-9)
Supplement: Supplementary file 6 — Reporting Summary [file 41467_2021_24917_MOESM6_ESM.pdf]

nature research

Corresponding author(s): Gage Crump & Steve Twigg

Last updated by author(s): Jun 22, 2021

Reporting Summary

Nature Research wishes to improve the reproducibility of the work that we publish. This form provides structure for consistency and transparency in reporting. For further information on Nature Research policies, see our Editorial Policies and the Editorial Policy Checklist.

Statistics

For all statistical analyses, confirm that the following items are present in the figure legend, table legend, main text, or Methods section.

n/a	Confirmed	
☐	☒	The exact sample size (*n*) for each experimental group/condition, given as a discrete number and unit of measurement
☒	☐	A statement on whether measurements were taken from distinct samples or whether the same sample was measured repeatedly
☐	☒	The statistical test(s) used AND whether they are one- or two-sided<br>*Only common tests should be described solely by name; describe more complex techniques in the Methods section.*
☒	☐	A description of all covariates tested
☒	☐	A description of any assumptions or corrections, such as tests of normality and adjustment for multiple comparisons
☒	☐	A full description of the statistical parameters including central tendency (e.g. means) or other basic estimates (e.g. regression coefficient) AND variation (e.g. standard deviation) or associated estimates of uncertainty (e.g. confidence intervals)
☐	☒	For null hypothesis testing, the test statistic (e.g. *F*, *t*, *r*) with confidence intervals, effect sizes, degrees of freedom and *P* value noted<br>*Give P values as exact values whenever suitable.*
☒	☐	For Bayesian analysis, information on the choice of priors and Markov chain Monte Carlo settings
☒	☐	For hierarchical and complex designs, identification of the appropriate level for tests and full reporting of outcomes
☒	☐	Estimates of effect sizes (e.g. Cohen's *d*, Pearson's *r*), indicating how they were calculated

Our web collection on statistics for biologists contains articles on many of the points above.

Software and code

Policy information about availability of computer code

Data collection	No code was used for data collection.
Data analysis	Quality control of raw reads - FastQC (0.11.7), FastQ Screen (0.7.0) and MultiQC (0.9). Singlecell RNA-sequencing data were processed and quantified using Cell Ranger (10X Genomics) version 2.1.1, aligning to the mm10 mouse transcriptome. Data analysis was performed with Seurat version 3.2.0. Cell–cell communication analysis was performed using CellphoneDB v2.1.4. Immunofluorescence analysis used Zeiss software: ZEN 2011 SP7 FP2 (black) Version 14.0.16.201.

For manuscripts utilizing custom algorithms or software that are central to the research but not yet described in published literature, software must be made available to editors and reviewers. We strongly encourage code deposition in a community repository (e.g. GitHub). See the Nature Research guidelines for submitting code & software for further information.

Data

Policy information about availability of data

All manuscripts must include a data availability statement. This statement should provide the following information, where applicable:

- Accession codes, unique identifiers, or web links for publicly available datasets
- A list of figures that have associated raw data
- A description of any restrictions on data availability

The raw and processed RNAseq data are available in the GEO repository, accession: GSE163693 (https://www.ncbi.nlm.nih.gov/geo/query/acc.cgi?acc=GSE163693). All other data are included in the article and Supplementary Information or available from the corresponding authors upon reasonable request.

Field-specific reporting

Please select the one below that is the best fit for your research. If you are not sure, read the appropriate sections before making your selection.

- [x] Life sciences
- [ ] Behavioural & social sciences
- [ ] Ecological, evolutionary & environmental sciences

For a reference copy of the document with all sections, see nature.com/documents/nr-reporting-summary-flat.pdf

Life sciences study design

All studies must disclose on these points even when the disclosure is negative.

Sample size	Sample sizes were deemed sufficient if the signal was replicated in a minimum of three independent animals. For the localization experiments at least three embryos were evaluated for every probe in order to confirm the consistency of expression patterns. The single cell analysis was carried out on at least 10 coronal sutures for each time point. All analyses for Twist1/Tcf12 mutant analyses were performed at least twice in two independent animals. Scx-GFP experiments were performed in replicates. Six2-CreERT2 experiments were performed in triplicate. Six2-Cre experiments were performed in quadruplicate.
Data exclusions	Cells with high mitochondria content, low UMI/ gene counts were removed to eliminate biased clustering, as were contaminating red blood cells.
Replication	Only probes that were reproducible across three embryos were included. Sutures from at least 5 different embryos were used for single cell analysis. At E17.5 single cell libraries were also prepared separately in 3 batches as a technical control. All experiments on Twist1/Tcf12 mutants was performed at least twice per probe. All experiments for Twist1/Tcf12 expression analysis and single cell validation were successfully replicated.
Randomization	No randomization was required as only wildtype conditions were interrogated.
Blinding	Most experiments only included wild type conditions and blinding was therefore not relevant. Similarly, for Twist1/Tcf12 experiments, we genotyped animals prior to in situ experiments to ensure that the correct genotypes were analyzed in combination and that control embryos were included in the same batch of experiments.

Reporting for specific materials, systems and methods

We require information from authors about some types of materials, experimental systems and methods used in many studies. Here, indicate whether each material, system or method listed is relevant to your study. If you are not sure if a list item applies to your research, read the appropriate section before selecting a response.

Materials & experimental systems

n/a	Involved in the study	
[ ]	[x]	Antibodies
[x]	[ ]	Eukaryotic cell lines
[x]	[ ]	Palaeontology and archaeology
[ ]	[x]	Animals and other organisms
[x]	[ ]	Human research participants
[x]	[ ]	Clinical data
[x]	[ ]	Dual use research of concern

Methods

n/a	Involved in the study	
[x]	[ ]	ChIP-seq
[ ]	[x]	Flow cytometry
[x]	[ ]	MRI-based neuroimaging

Antibodies

Antibodies used	Tcf7 (C63D9, Cell Signaling Technology); Cd200 (AF2724, R&D systems); Dmp1 (AF4386, R&D systems); Cx43 (3512, R&D systems); Crabp2 (10225-1-AP, Proteintech); Rabbit Anti-Sp7 (1:750; sc-22536-R, Santa Cruz Biotechnology), Chicken Anti-mCherry (1:500, NBP2-25158, Novus Biologicals), Donkey anti-Sheep IgG HRP (A16041, Roche), Donkey anti-goat IgG-HRP (sc-2020, Santa Cruz Biotechnology), Donkey anti-Rat IgG HRP (A18739, Thermo Fisher Scientific), Alexa Fluor 488, 555 and 647 secondary antibodies (A21206 or A11015, A21432, A31573; Thermo Fisher Scientific)
Validation	All antibodies are validated for their purpose by the suppliers: TCf7 - https://www.cellsignal.co.uk/products/primary-antibodies/tcf1-tcf7-c63d9-rabbit-mab/2203; CD200 - https://www.rndsystems.com/products/human-mouse-rat-CD200-antibody_af2724; DMP1 - https://www.rndsystems.com/products/mouse-dmp-1-antibody_af4386; Cx43 - https://www.cellsignal.co.uk/products/primary-antibodies/connexin-43-antibody/3512; CRABP22 - https://www.ptglab.com/products/CRABP2-Antibody-10225-1-AP.htm

Animals and other organisms

Policy information about studies involving animals; ARRIVE guidelines recommended for reporting animal research

Laboratory animals	Embryos from C57BL/6J mice were used for single cell experiments and RNA/immuno localization analysis. Twist1/Tcf12 mutant embryos were obtained from outcrosses of Twist1/Tcf12 mutant adults (alleles obtained from JAX) to wildtype C57BL/6J mice.<br><br>For Six2-Cre experiments, timed matings between Six2-Cre heterozygous males and Ai9 homozygous females were performed, and mothers were sacrificed at E16.5 to harvest embryos or 3 weeks after birth. Scx-GFP mice were screened for fluorescence using a fluorescent flashlight and sacrificed at 3 weeks. For tamoxifen treatments, newborn pups (P0) were obtained from crosses of Six2-CreERT2; R26-CAG-LSL-Sun1-sfGFP-myc males and Cre- negative females and injected with 10 uL of a 20 mg/mL tamoxifen solution dissolved in corn oil into the abdominal cavity. Swiss Webster foster mothers were used to avoid maternal rejection, and pups were sacrificed at P7.<br><br>All mice housed at the University of Southern California and University of Oxford were maintained on a 12:12 light-dark cycle (lights on at 6 PM) at a controlled temperature and humidity.
Wild animals	The study did not involve wild animals.
Field-collected samples	No field collection done.
Ethics oversight	All procedures were approved by the MRC Weatherall Institute of Molecular Medicine Ethics Committee, or the University of Southern California IACUC Committee and performed in accordance with the relevant guidelines and regulations.

Note that full information on the approval of the study protocol must also be provided in the manuscript.

Flow Cytometry

Plots

Confirm that:

- [x] The axis labels state the marker and fluorochrome used (e.g. CD4-FITC).
- [x] The axis scales are clearly visible. Include numbers along axes only for bottom left plot of group (a 'group' is an analysis of identical markers).
- [x] All plots are contour plots with outliers or pseudocolor plots.
- [x] A numerical value for number of cells or percentage (with statistics) is provided.

Methodology

Sample preparation	Flow cytometry was used to collect live cells. For E17.5 embryos coronal sutures were dissected out in ice cold PBS using a scalpel blade, isolating a strip containing the overlapping frontal and parietal bone fronts (which appears opaque compared to adjacent regions) and avoiding the most apical and basal aspects of the suture. Isolated sutural strips from embryos from two litters in 3 batches (batch 1, 10 sutures from litter 1; batch 2 and 3, 3 sutures each from litter 2) were cut into small fragments in HBBS and digested using Collagenase IV (Worthington, USA; final concentration in HBBS of 2 mg/mL) for 30 min. Dissociation was terminated with 2% Fetal Bovine Serum and cells were passed through a 0.35 µM filter (E15.5) or Pluri-strainer Mini 70 µm (E17.5; pluriSelect Life Science, Germany) prior to FACs.
Instrument	BD FACSAria Fusion; 100 µM nozzle
Software	BD FACSDiva 8
Cell population abundance	Approximately 50% of the starting material was collected as live individual cells.
Gating strategy	Standard gating procedures were used to remove debris, cell doublets and likely dead cells

- [x] Tick this box to confirm that a figure exemplifying the gating strategy is provided in the Supplementary Information.
